# Supplementary material for: Dysregulation of LINC00324 promotes poor prognosis in patients with glioma
Source: PLoS One. 2024 Mar 26;19(3):e0298055. doi: 10.1371/journal.pone.0298055 (PMC10965094; doi:10.1371/journal.pone.0298055)
Supplement: S1 Table — (DOCX) [file pone.0298055.s001.docx]

**Table S1.** **The correlation between LINC00324 expression and immune checkpoint-related genes^*^**

| Immune Checkpoints | Pearson correlation | 95%CI | *P* value |
| --- | --- | --- | --- |
| ADORA2A | -0.185 | -0.255 - -0.112 | 8.86e-07 |
| ARG1 | 0.254 | 0.183 - 0.322 | 9.09e-12 |
| BTLA | 0.373 | 0.308 - 0.435 | 1.59e-24 |
| CD274 | 0.431 | 0.369 - 0.490 | 5.48e-33 |
| CD276 | 0.526 | 0.470 - 0.578 | 5.15e-51 |
| CTLA4 | 0.378 | 0.312 - 0.439 | 4.27e-25 |
| EDNRB | -0.043 | -0.117 - 0.031 | 0.253 |
| HAVCR2 | 0.627 | 0.579 - 0.670 | 1.54e-77 |
| IDO1 | 0.384 | 0.319 - 0.445 | 5.63e-26 |
| IL4 | 0.175 | 0.102 - 0.246 | 3.17e-06 |
| IL10 | 0.591 | 0.541 - 0.637 | 4.80e-67 |
| IL12A | -0.014 | -0.088 - 0.060 | 0.716 |
| IL13 | -0.020 | -0.094 - 0.054 | 0.602 |
| KIR2DL1 | 0.247 | 0.176 - 0.315 | 3.79e-11 |
| KIR2DL3 | 0.262 | 0.192 - 0.331 | 1.66e-12 |
| LAG3 | 0.230 | 0.158 - 0.299 | 7.77e-10 |
| PDCD1 | 0.402 | 0.338 - 0.463 | 1.38e-28 |
| SLAMF7 | 0.465 | 0.405 - 0.521 | 9.64e-39 |
| TGFB1 | 0.567 | 0.514 - 0.615 | 1.37e-60 |
| TIGIT | 0.086 | 0.012 - 0.159 | 0.023 |
| VEGFA | 0.382 | 0.317 - 0.444 | 1.07e-25 |
| VEGFB | 0.274 | 0.204 - 0.341 | 1.61e-13 |
| VTCN1 | -0.024 | -0.098 - 0.050 | 0.526 |
| BTN3A1 | 0.461 | 0.400 - 0.517 | 5.11e-38 |
| BTN3A2 | 0.472 | 0.413 - 0.528 | 4.15e-40 |
| CCL5 | 0.542 | 0.488 - 0.592 | 1.17e-54 |
| CD27 | 0.257 | 0.186 - 0.325 | 5.39e-12 |
| CD28 | 0.423 | 0.361 - 0.482 | 8.68e-32 |
| CD40 | 0.604 | 0.554 - 0.649 | 1.36e-70 |
| CD40LG | 0.413 | 0.349 - 0.473 | 3.82e-30 |
| CD70 | 0.318 | 0.250 - 0.383 | 6.76e-18 |
| CD80 | 0.474 | 0.415 - 0.530 | 1.85e-40 |
| CX3CL1 | -0.307 | -0.373 - -0.238 | 9.24e-17 |
| CXCL10 | 0.580 | 0.529 - 0.628 | 3.45e-64 |
| CXCL9 | 0.483 | 0.424 - 0.537 | 4.74e-42 |
| ENTPD1 | 0.490 | 0.432 - 0.545 | 1.57e-43 |
| GZMA | 0.567 | 0.515 - 0.615 | 9.87e-61 |
| HMGB1 | 0.274 | 0.204 - 0.341 | 1.62e-13 |
| ICAM1 | 0.475 | 0.415 - 0.530 | 1.34e-40 |
| ICOS | 0.431 | 0.369 - 0.490 | 5.46e-33 |
| ICOSLG | 0.203 | 0.131 - 0.273 | 6.1e-08 |
| IFNA1 | -0.092 | -0.165 - -0.018 | 0.015 |
| IFNA2 | -0.174 | -0.245 - -0.101 | 3.60e-06 |
| IFNG | 0.237 | 0.165 - 0.305 | 2.41e-10 |
| IL1A | 0.453 | 0.392 - 0.510 | 1.11e-36 |
| IL1B | 0.342 | 0.274 - 0.405 | 1.48e-20 |
| IL2 | 0.217 | 0.146 - 0.287 | 6.30e-09 |
| IL2RA | 0.467 | 0.407 - 0.524 | 3.06e-39 |
| ITGB2 | 0.612 | 0.563 - 0.656 | 6.33e-73 |
| PRF1 | 0.499 | 0.441 - 0.552 | 3.37e-45 |
| SELP | 0.202 | 0.130 - 0.272 | 6.88e-08 |
| TLR4 | 0.154 | 0.081 - 0.225 | 4.44e-05 |
| TNF | 0.165 | 0.092 - 0.236 | 1.18e-05 |
| TNFSF4 | 0.343 | 0.276 - 0.407 | 9.46e-21 |
| TNFSF9 | -0.076 | -0.149 - -0.001 | 0.046 |
| TNFRSF4 | 0.430 | 0.367 - 0.488 | 9.26e-33 |
| TNFRSF9 | 0.320 | 0.252 - 0.385 | 3.87e-18 |
| TNFRSF14 | 0.491 | 0.432 - 0.545 | 1.31e-43 |
| TNFRSF18 | 0.248 | 0.177 - 0.316 | 2.98e-11 |

^*^ CI, confidence interval
